# Severe Illness Associated with Eating Mushroom-Containing Chocolate Products — United States, January–October 2024

**DOI:** 10.15585/mmwr.mm7513a2

**Published:** 2026-04-09

**Authors:** Jelonia T. Rumph, Andrea Winquist, Alyssa N. Troeschel, Skylar Pulver, Amy Helene Schnall, Joseph Ebersole, Michael Yeh, Brooke Burt, Sharon L. Federman, Karl Klontz, Catherine Dasenbrock, Heather L. Leahy, Shane Brady, Haylea Stuteville, Ketki Patel, Johnni Daniel, Arthur Chang

**Affiliations:** ^1^Epidemic Intelligence Service, Division of Workforce Development, CDC; ^2^Division of Environmental Health Science and Practice, National Center for Environmental Health, CDC; ^3^U.S. Public Health Service Commissioned Corps, Rockville, Maryland; ^4^Office of the Coordinated Outbreak Response, Evaluation & Emergency Preparedness, Human Foods Program, Food and Drug Administration, College Park, Maryland; ^5^Office of Surveillance Strategy and Risk Prioritization, Human Foods Program, Food and Drug Administration, College Park, Maryland; ^6^National Forensic Chemistry Center, Office of Specialty Laboratories and Enforcement Support, Office of the Chief Scientist, Office of the Commissioner, Food and Drug Administration, Cincinnati, Ohio; ^7^Arizona Department of Health Services; ^8^Maricopa County Department of Public Health; Phoenix, Arizona; ^9^Texas Department of State Health Services.

**Summary**

**What is already known about this topic?**

Edible products that contain small doses of psychoactive substances have been known to cause serious or life-threatening illnesses. In late spring 2024, CDC was alerted to an outbreak of illness potentially associated with eating mushroom-containing microdosing products.

**What is added by this report?**

A national investigation identified 180 cases of severe illness in 34 states associated with eating Diamond Shruumz or other mushroom-containing chocolate products. Among cases with available outcome data, 43.7% of persons required hospitalization, 23.2% required intensive care unit admission, 17.5% required endotracheal intubation, and 1.1% died. Severe outcomes were most common among persons who ate Diamond Shruumz chocolate bars, and outcomes worsened with larger amounts eaten. Diamond Shruumz products have since been recalled.

**What are the implications for public health practice?**

Consumers should be aware that microdosing psychedelic products can cause severe illness or death and that recalled products should not be sold, purchased, or eaten.

## Abstract

In late spring 2024, CDC was alerted to an outbreak of poisoning potentially associated with eating Diamond Shruumz microdosing chocolate bars. Diamond Shruumz microdosing chocolate bars are edible products designed so that small doses of mushroom-derived psychoactive compounds and other psychoactive ingredients can be eaten in a presectioned serving. In response to this alert, CDC and the Food and Drug Administration coordinated a nationwide outbreak investigation to characterize the potential poisonings associated with eating Diamond Shruumz microdosing chocolate bars. A case of poisoning was defined as an illness with moderate or major clinical effects (i.e., symptoms) as defined by America’s Poison Centers in a person who ate any Diamond Shruumz product or another mushroom-containing chocolate product during January–October 2024. In total, 180 cases were reported in 34 states. Among these, 73 persons were hospitalized, including 38 persons who required intensive care unit (ICU) admission, 29 who required endotracheal intubation, and two deaths. Eating Diamond Shruumz chocolate bars was associated with higher odds of hospitalization (odds ratio [OR] = 3.29; 95% CI = 1.51–7.40), ICU admission (OR = 6.30; 95% CI = 2.17–22.6), seizures (OR = 8.45; 95% CI = 3.00–27.9), and endotracheal intubation (OR = 8.04; 95% CI = 2.24–44.2), compared with eating other mushroom-containing chocolate products. Eating larger amounts of Diamond Shruumz chocolate bars was associated with an increased likelihood of hospitalization, ICU admission, and endotracheal intubation (p-value for trend tests [p-trend] = 0.023, 0.004, and <0.001, respectively). Diamond Shruumz products were recalled, and the public was advised not to eat, sell, or serve any Diamond Shruumz products and to discard any Diamond Shruumz products previously purchased. Testing of some Diamond Shruumz products identified substances present in psychoactive mushrooms, including muscimol, psilocin (a Schedule I controlled substance), kavalactones, and other substances in some, but not all, tested products. Consumers should be aware of the poisoning risk associated with eating Diamond Shruumz products and other mushroom-containing microdosing chocolate products due to variability in ingredient composition, the absence of standardized regulatory oversight for sampling and testing finished products, and the potential toxicity of compounds intended to produce psychoactive effects.

## Investigation and Results

In spring 2024, CDC was notified by America’s Poison Centers and the Arizona Department of Health Services (ADHS) of reports of four persons who had severe illness with symptoms including seizures or central nervous system (CNS) depression and had eaten Diamond Shruumz mushroom-containing microdosing chocolate bars (*1*). In response, CDC collaborated with the Food and Drug Administration (FDA) to initiate a national outbreak investigation, which led to identification of 180 cases in 34 states, including the four cases originally identified by ADHS and America’s Poison Centers. These data were previously reported (*1*). The ADHS investigation examined the four June 2024 Arizona cases of illness among persons who ate Diamond Shruumz chocolate bars (*1*). The resulting national investigation examined cases of illness among all U.S. persons who ate 1) any Diamond Shruumz products (including chocolate bars, gummies, and cones [products that resemble ice cream cones]) or 2) any other brands of mushroom-containing chocolate products during January–October 2024 across the United States (*2*,*3*). The national investigation evaluated the clinical characteristics and indicators of illness severity related to type and quantity of product eaten, using data from the National Poison Data System (NPDS), the data warehouse for the nation’s 53 poison centers, as well as compiling and storing reports to poison centers, FDA, health departments, and health care providers. This report describes the findings from that investigation.

### Data Source

During June–July 2024, after notification of the four Arizona poisoning cases identified by ADHS and America’s Poison Centers among persons who ate Diamond Shruumz chocolate bars, CDC reviewed data from NPDS for all reported cases nationwide. This review of NPDS data confirmed cases of severe illness in persons who ate Diamond Shruumz chocolate bars and identified new cases among persons who ate gummies or cones also branded as Diamond Shruumz. This review also identified additional cases of severe illness among persons who ate mushroom-containing chocolate products manufactured by other and unknown brands. CDC then collaborated with the Council of State and Territorial Epidemiologists to develop a questionnaire to collect information on the demographic characteristics, microdosing products eaten, signs and symptoms of illness, and types of health care received among persons with poisoning cases. Health departments completed the questionnaire using information obtained from participants (or their proxies), clinicians, and medical records.

### Case Definition and Ascertainment

A case of poisoning was defined as an illness characterized by moderate^†^ or major^§^ symptoms, including death, in a person who ate any Diamond Shruumz product (i.e., chocolate bars, gummies, or cones) or another brand of mushroom-containing chocolate product during January 1–October 11, 2024 (*2*). States used a standardized questionnaire to collect information about cases identified from any reporting source (e.g., poison centers, NPDS, FDA, or cases directly reported by affected persons or health care providers, including hospitals).

### Analyses

A descriptive analysis of cases was conducted, including demographic characteristics, clinical signs and symptoms, and products eaten. To ascertain the associations between eating specific types of products and indicators of severe illness (i.e., occurrence of seizures, emergency department or urgent care [ED/UC] visits, hospitalization, intensive care unit [ICU] admission, and endotracheal intubation), persons who ate only mushroom-containing chocolate products other than those branded as Diamond Shruumz were considered the referent group. Comparator groups included persons who ate Diamond Shruumz chocolate bars only, Diamond Shruumz gummies only, Diamond Shruumz cones only, and all other combinations (e.g., combined eating of any Diamond Shruumz product and any other mushroom-containing chocolate product). The Cochran-Armitage test was used to examine trends in the proportion of persons with indicators of severe illness across increasing amounts of Diamond Shruumz chocolate bars eaten; p<0.05 was considered statistically significant. All analyses were performed using SAS (version 9.4; SAS Institute). This activity was reviewed by CDC, deemed not research, and was conducted consistent with applicable federal law and CDC policy.^¶^

### Characteristics of Persons with Poisoning Cases

As previously reported, in addition to the initial four Arizona cases, an additional 176 U.S. cases were identified, for a total of 180 cases among 179 persons in 34 states (one person developed moderate or major clinical symptoms on two separate occasions, and each event was considered a separate case) (*1*). Illness onset occurred during January 1–October 11, 2024 (Supplementary Figure 1) (Supplementary Figure 2).

Among the 178 cases with available demographic information, 81 (46%) occurred among adults aged 18–29 years, and 99 (59%) of 168 cases with available information on sex occurred among males (Table 1). Among the cases for which health outcome information was available, 150 (87%) involved care in an ED/UC; 73 (44%) of poisoning events required hospitalization, 38 (23%) required ICU admission, and 29 (18%) required endotracheal intubation. The three most frequently reported signs and symptoms were confusion (66%), drowsiness (47%), and agitation (45%); 29% of cases involved seizures. Two (1.1%) deaths were noted as being associated with the outbreak.

**TABLE 1:** Characteristics of persons with moderate or major symptoms after eating a Diamond Shruumz or another mushroom-containing chocolate product (N = 180) — United States, January 1–October 11, 2024*

Characteristic (no. with available information)^†^	No. (%)
**Age group, yrs (n = 178)**	
<18	24 (13.5)
18–29	81 (45.5)
30–39	39 (21.9)
40–49	20 (11.2)
≥50	14 (7.9)
**Sex (n = 168)**	
Female	69 (41.1)
Male	99 (58.9)
**Race and ethnicity (n = 125)**	
Black or African American, NH	18 (14.4)
Hispanic or Latino	13 (10.4)
White, NH	89 (71.2)
Other (including multiracial)	5 (4.0)
**Signs and symptoms**	
Date of first onset of signs and symptoms, median (range) (n = 155)	Jun 8 (Jan 6–Sep 12)
Confusion (n = 157)	104 (66.2)
Drowsiness or difficulty staying awake (n = 150)	71 (47.3)
Agitation (n = 155)	69 (44.5)
Loss of consciousness (n = 155)	67 (43.2)
Hallucinations or delusions (n = 137)	59 (43.1)
Nausea (n = 144)	58 (40.3)
Feeling of a fast or irregular heartbeat (n = 137)	56 (40.9)
Tachycardia (n = 130)	53 (40.8)
Vomiting (n = 153)	52 (40.0)
Hypertension (n = 129)	46 (35.7)
Seizure (n = 144)	41 (28.5)
Shortness of breath (n = 137)	32 (23.4)
**Health outcomes**	
ED/UC visit (n = 172)	150 (87.2)
Hospitalized (n = 167)	73 (43.7)
Admitted to ICU (n = 164)	38 (23.2)
Received endotracheal intubation (n = 166)	29 (17.5)
Died (n = 174)	2 (1.1)
**Length of hospitalization, days, mean (SD) (67)**	2.2 (4.4)
Median (range)	1.0 (<1–33.0)
**Duration of endotracheal intubation, mean (SD) (27)**	1.6 (1.1)
Median (range)	1.0 (<1–4.0)
**Types of products eaten^§^**	
Any Diamond Shruumz product (n = 148)	118 (79.7)
Any Diamond Shruumz chocolate bar^¶^ (n = 130)	85 (65.4)
Any Diamond Shruumz gummies** (n = 115)	14 (12.2)
Any Diamond Shruumz cone^†† ^(n = 118)	11 (9.3)
Any other chocolate product^§§^ (n = 151)	64 (42.4)^¶¶^
**Interval from eating product to symptom onset (minutes) (n = 96)**	
Median (IQR)	90 (30–180)
Range	0 (10–109)

**Abbreviations**: ED/UC = emergency department/urgent care; ICU = intensive care unit; NH = non-Hispanic.

* One person developed moderate or major symptoms twice; therefore, a total of 180 cases occurred among 179 persons.

^†^ The denominator represents the number of cases for which information for that particular variable was available. Percentages are based on denominators excluding missing data.

^§^ Reports of products eaten are not mutually exclusive; some persons with cases reported eating more than one type of product (i.e., 166 persons ate one type of product, 10 persons ate two types of products, and one person each ate three types of products, four types of products, five types of products, or six types of products).

^¶^ Ate at least one of the available flavors of Diamond Shruumz chocolate bars (i.e., dark chocolate, fruity cereal, cinnamon, birthday cake, cookie butter, and cookies and cream).

** Ate at least one of the available flavors of Diamond Shruumz gummies (i.e., strawberry kiwi, grape lemonade, sour apple peach, rainbow, Hawaiian punch, blue razz watermelon, blue razz euphoria, watermelon wonderland, radical rainbow, lucid lemon-lime, and peach paradise).

^††^ Ate at least one of the available flavors of Diamond Shruumz cones (i.e., double chocolate chip, mint chocolate chip, sprinkles, cookies and cream, and strawberry cheesecake).

^§§^ Chocolate products other than Diamond Shruumz included Awaken Superfood White Chocolate, Fusion, Hixotic, Lucid Journeys, Magic Kingdom, Megadose Rize Milk Crunch, Moom, Mushroom Lyfe, Polka Dot (Magic Belgium Milk Chocolate Mushroom Candy, Belgian Chocolate Bar, Magic Chocolate, Magic Mushroom, and Belgian Chocolate Reese), Punch edible, Stoned, Sunday Bar, Super Smashed (Mushrooms and Shrooms Magic), Tre House Magic Mushroom, Willy Wonky Mushroom Chocolate Bar, Wonderbar, mmelt Magic Mushroom Chocolate Bars, and Wonderland Happy Dots Proprietary Magical Blend. The brand was not specified for 36 cases.

^¶¶^ This category includes two persons who consumed both Diamond Shruumz and non–Diamond Shruumz products.

### Types of Products Eaten and Indicators of Severe Illness

Among all 180 cases, 118 (66%) involved persons who had eaten any Diamond Shruumz product, including 85 (72%) who had eaten a Diamond Shruumz chocolate bar and 25 (21%) who had eaten Diamond Shruumz gummies (14) or cones (11) (Supplementary Table). Approximately one third of cases (62; 34%) involved eating a chocolate product not branded as Diamond Shruumz, and eating both Diamond Shruumz and other brands of mushroom-containing chocolate products was reported in an additional two cases (1.1%). Among 62 persons who reportedly consumed only other mushroom-containing chocolate products, a specific brand was named for 26 (42%). The median interval between eating a product and onset of first symptoms was 90 minutes (IQR = 30–180 minutes) (Table 1).

Cases among persons who had eaten only Diamond Shruumz chocolate bars were significantly more likely to involve seizures (odds ratio [OR] = 8.45), hospitalization (OR = 3.29), ICU admission (OR = 6.30), and endotracheal intubation (OR = 8.04) than were cases among persons who had eaten only other chocolate products (Table 2). The prevalences of hospitalization, ICU admission, and endotracheal intubation significantly increased with increasing amounts of Diamond Shruumz chocolate bars eaten (p-trend = 0.024, 0.004, and <0.001, respectively) (Table 3).

**TABLE 2:** Associations between type of products eaten and indicators of severe illness among persons with moderate or major symptoms — United States, January 1–October 11, 2024

Outcome and types of products eaten (no. with available information)*	No. with outcome (column %)		OR (95% CI)
	Yes	No	
**Seizures (n = 41/137)**			
Diamond Shruumz chocolate bars only	30 (73.2)	29 (30.2)	8.45 (3.00–27.9)
Diamond Shruumz gummies only	3 (7.3)	5 (5.2)	4.82 (0.60–33.5)
Diamond Shruumz cones only	1 (2.4)	7 (7.3)	1.19 (0.02–12.5)
All other combinations	1 (2.4)	5 (5.2)	1.65 (0.03–19.1)
Any other chocolate product only	6 (14.6)	50 (52.1)	Ref**^†^**
**Total**	**41 (100.0)**	**96 (100.0)**	**—**
**Emergency department or urgent care center visit (n = 143/164)**			
Diamond Shruumz chocolate bars only	69 (48.3)	10 (47.6)	NC
Diamond Shruumz gummies only	4 (2.8)	4 (19.1)	NC
Diamond Shruumz cones only	5 (3.5)	3 (14.3)	NC
All other combinations	4 (2.8)	4 (19.1)	NC
Any other chocolate product only	61§ (42.7)^§^	0 (—)	Ref**^†^**
**Total**	**143 (100.0)**	**21 (100.0)**	**—**
**Hospitalization (n = 71/159)**			
Diamond Shruumz chocolate bars only	44 (62.0)	34 (38.6)	3.29 (1.51–7.40)
Diamond Shruumz gummies only	5 (7.0)	3 (3.4)	4.16 (0.71–30.0)
Diamond Shruumz cones only	3 (4.2)	5 (5.7)	1.53 (0.21–8.96)
All other combinations	3 (4.2)	5 (5.7)	1.53 (0.21–8.96)
Any other chocolate product only	16 (22.5)	41 (46.6)	Ref**^†^**
**Total**	**71 (100.0)**	**88 (100.0)**	**—**
**ICU admission (n = 37/156)**			
Diamond Shruumz chocolate bars only	30 (81.1)	47 (39.5)	6.30 (2.17–22.6)
Diamond Shruumz gummies only	2 (5.4)	6 (5.0)	3.25 (0.26–26.4)
Diamond Shruumz cones only	0 (—)	8 (6.7)	NC
All other combinations	0 (—)	8 (6.7)	NC
Any other chocolate product only	5 (13.5)	50 (42.0)	Ref**^†^**
**Total**	**37 (100.0)**	**119 (100.0)**	**—**
**Endotracheal intubation (n = 29/158)**			
Diamond Shruumz chocolate bars only	24 (82.8)	53 (41.1)	8.04 (2.24–44.2)
Diamond Shruumz gummies only	2 (6.9)	6 (4.7)	5.73 (0.40–61.8)
Diamond Shruumz cones only	0 (—)	8 (6.2)	NC
All other combinations	0 (—)	8 (6.2)	NC
Any other chocolate product only	3 (10.3)	54 (41.9)	Ref**^†^**
**Total**	**29 (100.0)**	**129 (100.0)**	—

**Abbreviations:** ICU = intensive care unit; NC = not calculable; OR = odds ratio; Ref = referent group.

* Cases occurred among persons who ate Diamond Shruumz products (chocolate bars, gummies, or cones) or other brands of mushroom-containing chocolate products. Cases were excluded if eating Diamond Shruumz products was reported without specification of product type. If eating one specific product type was reported, but information was missing for other product types, no exposure to the unknown products was assumed. If Diamond Shruumz products and another brand of chocolate products were eaten, cases were coded as all other combinations. Some ORs were not calculable due to zero counts in some cells.

^†^ The Ref for this analysis comprised participants who reported eating only another brand of chocolate bar (i.e., no Diamond Shruumz products were reportedly eaten). However, some participants who might not have known the brand of mushroom-containing chocolate bar they ate might have selected this option; therefore, the Ref might contain persons who ate Diamond Shruumz products.

^§^ A total of 62 persons consumed another brand of mushroom-containing chocolate products only, but only 61 of these persons also had emergency department or urgent care outcome data.

**TABLE 3:** Number of persons* with indicators of severe illness among those with moderate or major symptoms^†^ after eating Diamond Shruumz chocolate bars, by number of pieces eaten — United States, January 1–October 11, 2024

Outcome level	No. of pieces eaten, no. (column %)				p-value for trend^§^
	0.5–3	4–6	7–10	>10	
**Seizure**					
Yes	2 (25)	3 (60)	6 (85.7)	10 (47.6)	0.43
No	6 (75)	2 (40)	1 (14.3)	11 (52.4)	
**Emergency department or urgent care center visit**					
Yes	3 (33.3)	5 (83.3)	16 (100)	24 (88.9)	<0.001
No	6 (66.7)	1 (16.7)	0 (0)	3 (11.1)	
**Hospitalization**					
Yes	3 (33.3)	2 (33.3)	8 (57.1)	19 (70.4)	0.024
No	6 (66.7)	4 (66.7)	6 (42.9)	8 (29.6)	
**ICU admission**					
Yes	1 (11.1)	1 (16.7)	4 (28.6)	16 (59.3)	0.004
No	8 (88.9)	5 (83.3)	10 (71.4)	11 (40.7)	
**Endotracheal intubation**					
Yes	0 (0)	0 (0)	4 (28.6)	14 (51.9)	<0.001
No	9 (100)	6 (100)	10 (71.4)	13 (48.1)	

**Abbreviation:** ICU = intensive care unit.

* Analysis was restricted to persons who ate Diamond Shruumz chocolate bars. An entire Diamond Shruumz chocolate bar consists of 12–15 presectioned pieces with a total weight of 1.6 oz (43.4 g). Diamond Shruumz’s website suggested that eating two squares was a “starting dose for microdosing”; no maximum dose was listed.

^†^ Moderate symptoms are more pronounced, more prolonged, or characterized as having a more systemic nature than are minor symptoms (i.e., symptoms that are minimally bothersome to the patient and resolve rapidly with no residual disability or disfigurement). Usually, some form of treatment is indicated for moderate symptoms, but they are not life-threatening and do not result in any residual disability or disfigurement. Major symptoms are life-threatening or result in a substantial residual disability or disfigurement.

^§^ Cochran-Armitage trend test.

## Public Health Response

### Notification and Voluntary Product Recall

On June 7, 2024, FDA published a notification about the investigation of the cases (*3*). On June 12, CDC released a Health Alert Network advisory alerting clinicians of the outbreak (*2*), and on June 18, FDA contacted the brand owner, requesting a voluntary recall of all Diamond Shruumz products. The brand owner issued a nationwide recall for all Diamond Shruumz products (*3*). By July 16, state partners reported that some recalled products were still available at several smoke and vape shops, and FDA worked with the National Association of Convenience Stores and the National Smoke Shop Association to increase awareness of the recall. On July 20, FDA published, and subsequently updated, a list of Diamond Shruumz suppliers (*3*).

### Product Testing Results

Early in the investigation, FDA and state partners collected samples of Diamond Shruumz products from several sources, and FDA tested 54 samples at the National Forensic Chemistry Center. As of November 15, 2024, testing had identified chemicals found in psychoactive mushrooms (i.e., muscimol and psilocin; psilocin is a Schedule I controlled substance), the kava plant (i.e., kavalactones), a synthetic hallucinogen (i.e., acetylpsilocin), and a pharmaceutical (i.e., pregabalin) in various samples (*2*,*3*). FDA testing also detected muscimol and ibotenic acid in a raw ingredient that was reportedly used in manufacturing some Diamond Shruumz products (*3*). Different samples of the same product type and flavor contained different substances.

## Discussion

A nationwide investigation identified 180 cases of moderate or major illness in persons who ate Diamond Shruumz microdosing products and other mushroom-containing chocolate products (*1*). The findings in this report indicate that Diamond Shruumz chocolate bars were more likely to be associated with indicators of severe illness, especially at higher levels of consumption, than were non–Diamond Shruumz chocolate products.

Multiple substances identified in Diamond Shruumz products might have contributed to severe illness through CNS modulatory neurotransmitters (*4*–*7*). Muscimol and certain kavalactones act on the GABA-A receptor and are associated with CNS depression (*7*,*8*). Seizures have been reported in cases of poisoning from *Amanita muscaria* (a mushroom containing muscimol and ibotenic acid), pregabalin, and tryptamines (e.g., psilocin, psilocybin, and dimethyltryptamine) (*5*,*6*,*8*). Because the products and patient symptoms varied widely, the exact pathway leading to severe illness in these cases remains unclear.

Approximately one third of all cases occurred among persons who reportedly ate only other mushroom-containing chocolate products, suggesting that the risk for moderate or major illness might not be limited to Diamond Shruumz products, although some consumers might have misreported eating a different chocolate product when they had actually eaten a Diamond Shruumz product. In addition, each person who ate only other mushroom-containing chocolate products sought emergency department care, possibly reflecting differential case finding rather than increased illness severity.

### Limitations

The findings in this report are subject to at least two limitations. First, this investigation likely underestimates the incidence of mushroom-containing microdosing product poisonings in the United States because persons might be reluctant to report eating these products, particularly persons who had less severe outcomes. In addition, the case definition only included reports of illness after eating Diamond Shruumz products of any type (i.e., chocolate, gummies, or cones) as well as other brands of mushroom-containing chocolate products, excluding other forms of mushroom-containing edible products, such as gummies, manufactured by other brands. A previous report by the Blue Ridge Poison Center in Charlottesville, Virginia, described five persons who sought medical attention after eating different mushroom gummy brands (*9*). Although the exact brands eaten in the Virginia investigation were unavailable for testing, analysis of several brands purchased by investigators revealed the presence of potentially harmful undisclosed ingredients and Schedule I drugs (i.e., psilocin and psilocybin) (*9*). Second, this investigation was limited to the collection of information from persons experiencing moderate or major illness after eating specific microdosing products; therefore, no comparison group of persons who did not eat these products was available for analysis, and comparisons were among persons who ate at least one microdosing product. However, the detailed information available on product types and amounts enabled dose-response analyses and the identification of product types that were potentially associated with more severe outcomes.

### Implications for Public Health Practice

The findings in this report highlight the potential risks associated with eating microdosing mushroom products. On December 18, 2024, FDA issued an alert declaring *Amanita muscaria*, its extracts, and certain constituents (e.g., muscimol and ibotenic acid) are not authorized for use as ingredients in conventional food (i.e., food additives) (*10*). Consumers should be aware of the potential for severe health risks associated with eating microdosing products harboring ingredients not suitable for human consumption. Consumers and clinicians can contact America’s Poison Centers for poison triage services by calling the Poison Help Line at 1-800-222-1222 or through the PoisonHelp.org website.

## Footnotes

* These senior authors contributed equally to this report.

† Moderate clinical effects are symptoms resulting from an exposure that are more pronounced, more prolonged, or of a more systemic nature than are minor symptoms (i.e., symptoms that are minimally bothersome to the patient and resolve rapidly with no residual disability or disfigurement). Usually, some form of treatment is indicated for moderate symptoms, but moderate symptoms are not life-threatening and do not result in a residual disability or disfigurement. Examples include hypotension that resolves with treatment, isolated brief seizures that resolve spontaneously or rapidly with treatment, and hypoglycemia with confusion.

§ Major symptoms are life-threatening or result in a substantial residual disability or disfigurement. Examples include status epilepticus, cardiovascular instability, and coma with hypotension.

¶ 45 C.F.R. part 46, 21 C.F.R. part 56; 42 U.S.C. Sect. 241(d); 5 U.S.C. Sect. 552a; 44 U.S.C. Sect. 3501 et seq.
